# Global Emergency Medicine Fellowships: Survey of Curricula and Pre-Fellowship Experiences

**DOI:** 10.5811/westjem.2020.11.49008

**Published:** 2020-12-19

**Authors:** Elise Klesick, Wael Hakmeh

**Affiliations:** Western Michigan University School of Medicine, Department of Emergency Medicine, Kalamazoo, Michigan

## Abstract

**Introduction:**

Lack of accreditation requirements affords global emergency medicine (GEM) fellowships the flexibility to customize curricula and content. A paucity of literature exists describing the state of GEM fellowship programs. We describe the current state of GEM fellowship curricula including which components are commonly included, and highlighting areas of higher variability.

**Methods:**

We identified GEM fellowships and invited them to participate in a web-based survey. Descriptive statistical analysis was performed.

**Results:**

Of the 46 fellowship programs invited to participate, 24 responded; one duplicate response and one subspecialty program were excluded. The 22 remaining programs were included in the analysis. Nineteen programs (86%) offer a Masters in Public Health (MPH) and 36% require an MPH to graduate. Additionally, 13 programs (59%) offered graduate degrees other than MPH. Fellows average 61 clinical hours per month (95% confidence interval, 53–68). Time spent overseas varies widely, with the minimum required time ranging from 2–28 weeks (median 8 weeks; interquartile range [IQR] 6,16) over the course of the fellowship. The majority of programs offer courses in tropical medicine (range 2–24 weeks, median 4 weeks) and the Health Emergencies in Large Populations course. Only 32% of programs reported offering formal ultrasound training. Fellows averaged 1.3 research projects prior to fellowship and median of 2.5 during fellowship (IQR 1,3). While the majority of GEM fellowship graduates worked at US academic centers (59%), 24% worked in US community hospitals, 9% worked for non-profit organizations, and 9% worked internationally in clinical practice.

**Conclusion:**

Our results highlight the wide variability of curricular content and experiences offered by GEM fellowships.

## INTRODUCTION

### Background

Global emergency medicine (GEM) is a broad and growing subspecialty within emergency medicine (EM). GEM is a diverse field that includes clinicians involved in tropical medicine, trauma, disaster response, and epidemiology, as well as researchers, public health experts, and professionals working to improve global health systems and policies. Many GEM clinicians are also involved in training physicians and other providers to advance EM in other countries.^1^ The number of GEM fellowships has greatly increased over the past 20 years. A survey in the early 2000s identified eight such fellowships, yet the Society for Academic Emergency Medicine (SAEM) currently lists 42 global international EM fellowships.^2^, ^3^ Because GEM fellowships are not accredited by bodies such as the Accreditation Council for Graduate Medical Education (ACGME), programs have the ability to customize curricula based on each fellowship’s resources, geographic advantages or limitations, and connections with international colleagues and nonprofit organizations. Little is currently known about the diversity and variation currently present among GEM curricula.

### Importance

In the 1990s, as EM continued to grow as a specialty internationally, fellowship training was identified as a means of equipping physicians to help develop EM residency programs overseas.^4^ Multiple models for GEM fellowship curricula have been proposed and tested since then,^5^,^6^ including a basic curricular framework that features seven core topics. Beran et al. describe how one fellowship used the seven core curricular elements model to redesign its program.^7^

By the early 2000s, there were eight GEM fellowships.^2^ All offered MPH degrees and offered fellows a variety of opportunities to travel and develop academic projects. Fellowship graduates were found to work primarily in academic settings, although all graduates reported continued involvement in international health.^2^ In 2011, GEM fellowships were surveyed and graded on how well each program covered six topics of interest.^8^ The study did not delve into what experiences fellows can expect, and curricula are likely to have evolved in the years since.

While there are a number of fellowship programs providing additional training to emergency physicians interested in global health, GEM is currently not an accredited subspecialty. Although lack of accreditation requirements provides programs flexibility in what experiences they can offer, it also means that there is no standardized core curriculum across all fellowships. There is a paucity of available literature describing expectations of GEM fellows before, during, and after fellowship.

### Goals of This Investigation

The purpose of this study was to describe the current curricula and experiences offered by GEM fellowship programs, identify what experiences are common or expected prior to GEM fellowship, and describe what careers graduates of these fellowships ultimately pursue.

## METHODS

### Study Design and Population

We used a cross-sectional survey of GEM fellowships. We developed the online survey using a nominal group technique with the following goals: 1) describe GEM fellowship curricula and experiences offered by GEM fellowship programs; 2) identify which experiences are common or expected prior to GEM fellowship; and 3) describe the career paths that GEM fellows pursue immediately post graduation. The survey was initially piloted on five emergency physicians (a department chair, a fellowship director, and three residents) and revised based on their feedback. The survey was converted into an electronic format on a university online platform. This research was approved by the applicable institutional review board.

We identified GEM programs using two publicly available databases,^3^,^9^ and additional programs were identified by internet searches and word of mouth. Additional identified programs were added until three weeks before the end of data collection.^9^,^10^ For a program to be included, fellowships had to accept EM residency graduates, be run either by a department of EM or have a track specifically designed for EM residency graduates, and the program had to be either currently active or active within the past three years. Programs were considered inactive and hence excluded if 1) the listed program director or coordinator reported via direct communication with us that the program was inactive, or 2) the institution did not have a webpage for the GEM fellowship and did not list the fellowship on its online list of fellowships offered. We excluded programs if they focused primarily on a specific niche within GEM (eg, pediatric GEM, or toxicology). Consent was obtained from all participants at the beginning of the survey.

**Population Health Research Capsule**

What do we already know about this issue?

Global emergency medicine (GEM) fellowships are not currently accredited, which allows variations in curricula. A survey in the early 2000s revealed all GEM fellowships offered a MPH, but curricula were otherwise diverse.

What was the research question?

What is the current status of GEM fellowships, including fellowship curricula, and pre- and post-fellowship experiences?

What was the major finding of the study?

All responding programs offer a masters degree and time to work overseas. The remainder of fellowship experiences are variable.

How does this improve population health?

This study informs physicians considering GEM fellowship of the diversity in curricula. Applicants are empowered to find the program and curricula that best support their career goals.

### Survey Content and Administration

The survey included multiple choice and free-text responses. Programs were asked demographic questions including number of fellows accepted annually, and length of fellowship. Participants were asked to disclose which advanced degrees were offered, and whether or not specific courses and training programs were included in fellowship curriculum. Participants were then asked to identify how many fellowship graduates went on to work in various settings.

We collected data between January–April 2019. Eligible program directors and coordinators were emailed invitations to participate along with a survey link. Nonresponders were sent reminder emails weekly for two weeks. A final personalized email invitation was sent to directors of nonresponding programs prior to terminating data collection. Due to difficulty ascertaining how many programs were active, nonresponding programs were contacted by email or phone. Programs were asked if they were still active, and if they had a fellow in the 2019–2020 academic year. We collected and managed survey responses using RedCap (Vanderbilt University, Nashville, TN) and sent them in aggregate to investigators with personal identifiers removed to maintain anonymity.

### Data Analysis

We performed descriptive analysis using SAS v9.4 (SAS Institute, Cary, NC). Categorical responses were reported as frequency (percentage), and numerical responses were reported as mean (standard deviation [SD]) or mean (interquartile range [IQR]).

## RESULTS

**Figure 1:**
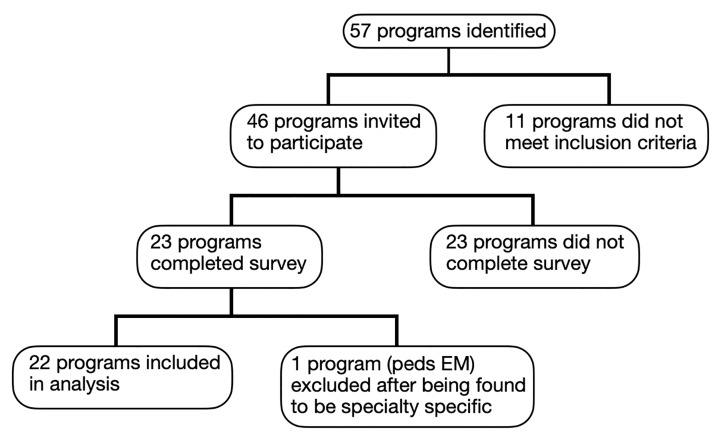
Global Emergency Medicine fellowship programs included. *EM*, emergency medicine.

We identified 57 GEM programs, of which 11 were excluded. (See Figure 1 for more details.) Seven programs were initially excluded due to being clearly inactive and therefore not meeting inclusion criteria. Three programs were excluded due to lack of an email address for a program director or coordinator. One program was excluded for having too narrow a focus (toxicology).

Ultimately, survey invitations were distributed to 67 fellowship directors and coordinators representing 46 programs; many programs listed only one contact person. Surveys were completed by 24 participants representing 23 fellowship programs. One response was omitted due to two responses from the same fellowship. (The response from the program coordinator was disregarded in favor of one completed by the program director.) An additional survey response was omitted due to the fellowship not meeting inclusion criteria (the fellowship was later found to be centered on pediatric EM). We included in the analysis results from the remaining 22 surveys.

There was difficulty in ascertaining which programs were definitely inactive. A total of 37 programs confirmed via phone or email that they were active. Three emails and three phone calls on different days went unanswered by the remaining nine fellowship programs, which raised concerns that they were likely also inactive despite being listed on various websites as being active. Twenty-nine programs indicated they had fellows. The overall response rate to all programs emailed was 22/45 (49%), but the response rate among programs that currently had fellows was 22/29 (76%).

Fifteen (68.2%) programs accepted one fellow per year, and seven (31.8%) accepted two fellows per year. One (4.6%) program was one year in length, while 21 (95.5%) programs were two years in length. Fellows averaged anywhere from 30–90 clinical hours per month, with an average of 61 hours (SD 13.5). The amount of time fellows spent outside the US varied widely, from a minimum of 2–28 weeks (median 8 weeks, IQR [6,16]), to a maximum of 8–52 weeks (median 24 weeks, IQR (15, 28).

Nineteen (86.4%) programs offered a Master of Public Health (MPH) degree, while only three (13.6%) programs did not, with 14 (63.6%) programs requiring a MPH degree by time of graduation. Thirteen programs (59.1%) offered other degrees, such as a Diploma of Tropical Medicine and Hygiene, Master of Business Administration, Master of Science, and Global Health Certificate.

Respondents were asked to report how many research experiences accepted fellows participated in during residency on average. Three (13.6%) respondents did not know. One (4.6%) respondent reported zero experiences during residency; 12 (54.6%) respondents reported one experience; four (18.2%) reported two experiences; and two (9.1%) reported three experiences. Regarding research during fellowship, five (22.7%) programs reported that fellows participated in 0–1 research experiences during fellowship, six (27.3%) reported two research experiences, seven (31.8%) reported three research experiences, two (9.1%) reported four research experiences, one (4.6%) reported five experiences, and one (4.6%) reported over 10 research experiences during fellowship.

Sixteen programs (73%) offered the Health Emergencies in Large Populations course, 11 (50%) a humanitarian response class, and one (5%) offered the American College of Emergency Physicians Emergency Medicine Basic Research Skills course. Seven programs dedicated time to point-of-care ultrasound training, with amount of time ranging from 2–100 hours per year. A tropical medicine course was offered by 14 programs (64%) spanning 2–24 weeks (median 4 weeks, IQR 3.5–10.5 weeks). Other program offerings included attendance at the African Federation for Emergency Medicine and International Federation for Emergency Medicine meetings, disaster medical assistance team training and deployments, humanitarian emergency and hospital disaster simulation, physician leadership courses, and access to the World Health Organization.

Finally, programs were asked to report how many recent graduates from their program were practicing in each of the following settings. Programs reported 59% of graduates working in US academic centers; 24% of graduates in US community practice settings, 9% for nonprofit agencies, and 9% in international clinical practice.

## DISCUSSION

This study demonstrates substantial variation in current GEM fellowship curricula. All participating fellowships offered advanced degrees, included international projects, and required clinical responsibilities. Areas of diversity included the specific degrees offered, amount of time devoted to clinical duties, travel, ultrasound training, and whether or not specific courses and conferences were included in fellowship curricula. Lack of accreditation permits GEM fellowship programs to customize their curricular offerings to each program’s strengths. This lack of curricular consistency allows fellowship applicants to choose programs with curricula more tailored to their career goals. However, it also reflects a lack of standardized expectations within the growing field of GEM.

Our results are consistent with previous research that has established variability in fellowship curricula. There was no single experience offered by all responding programs, except for participation in an international project. Unlike Bledsoe et al.,^2^ who reported that all GEM fellowships offered MPH training, we found 13.6% of programs did not offer a public health degree. As in the early 2000s survey, all responding programs were one or two years in duration and accepted one or two fellows per year. The amount of time devoted to clinical duties, and amount of time fellows could work overseas were widely variable, with average clinical hours varying threefold, from 30–90 hours per month. Fellows could spend as little as two weeks or as many as 52 weeks overseas each year, depending on the program. Fellowship applicants could choose from a range of advanced degree offerings, with MPH and Diploma of Tropical Medicine and Hygiene being most commonly available.

The majority of responding programs reported incoming fellows having participated in one research project during residency. Fellows typically completed up to three research projects during fellowship. No prior studies have reported research expectations prior to and during GEM fellowship.

Consistent with previous data, we found that a majority of fellowship graduates (59%) went on to work in university-affiliated academic settings. An additional 24% of graduates were reported to be working primarily in US community practice settings, for a total of 83% having a primary workplace within a US hospital or academic system. We found that 14% were reported to be working elsewhere, split evenly between international clinical practice and nonprofit agencies such as the US Centers for Disease Control and Prevention. Bledsoe et al. found that 79.3% of graduates worked in academic settings, and the remaining 20% in community practice. The field of GEM has grown over the past 20 years, and our research studied more than twice as many programs and graduates (22 programs and 80 graduates).

Despite the wide range of curricular components, there have been some recent efforts within GEM toward increased consistency. We found that a uniform application platform and deadline for submission of GEM fellowship applications were supported by a majority of program directors and recent fellows. An agreement among GEM Fellowship Consortium (GEMFC) members now exists to not offer positions to prospective candidates until a designated “match” day in an attempt to maximize the ability of both prospective candidates and programs to identify the best match. While there has been some standardization around the administrative aspects of fellowship, the process of identifying key core elements that should be included in GEM fellowship is lacking, particularly given the lack of knowledge about the existing variability in curricula.

Most current fellows and recent graduates surveyed admitted to little knowledge of their fellowship curriculum prior to starting.^11^ The Global Emergency Medicine Fellowship Consortium and the International Federation of Emergency Medicine recently collaborated to review methods of assessing GEM fellows. They found inconsistent methods of assessing fellows and created a consensus framework for a more standardized and outcome-oriented evaluation process.^12^ While flexibility in curricula is important, more work is required to identify differences in curricula, the value of each course and experience to best identify whether a common denominator of curricula should exist across all GEM fellowships.

The reasons for variability in curricula are likely diverse. Each institution and fellowship leader brings different strengths and connections. There are likely financial reasons, as well. Emergency physicians in non-accredited fellowships are able to work as independent practitioners, and the revenue they generate may provide significant funding for fellowship programs.^13^

An unintended, interesting finding from our research was the surprising amount of difficulty in determining which fellowship programs are currently active. Outdated information online contributed to this problem. While 37 programs appeared to be active, 29 programs confirmed via phone or email that they had current or incoming fellows. After conclusion of the study, we reached out to the GEMFC; their most recent unofficial statistics for the 2019–2020 academic year showed 20 programs offering 26 slots (with five of the programs only accepting internal applicants).

Future directions for research include surveying GEM fellowship graduates to explore which aspects of their training were least and most helpful, and how fellowship has affected their career choices and opportunities.

## LIMITATIONS

Limitations of this study include poor response rate. The reason for this is likely multifactorial. There were multiple programs contacted that reported being currently inactive, indicating that some non-responding programs may also be inactive. Jacquet et al. reported similar limitations to their 2013 survey, indicating that lack of an accurate count of current fellowships and lack of contact information have been a long-term problem for GEM.^11^ The data collection period was outside the fellowship application season, when fellowship email addresses may not be monitored as closely. This lack of accurate fellowship information online may pose a problem for potential applicants as well. Potentially higher response rates from programs with more resources may contribute to selection bias and over-representation.

Although we elucidated what experiences are commonly offered by international EM fellowships, we did not ask whether many of these experiences are required for graduation. Fellows often have leeway to choose which experiences are important to their career development, and we anticipate even less concordance in graduation requirements across programs compared to curricular offerings.

## CONCLUSION

In summary, our results highlight the wide variety of experiences offered by global emergency medicine fellowships. While there are common offerings including an MPH, tropical medicine training, and Health Emergencies in Large Populations course, no experience was offered by all responding programs except for international travel. While many similarities exist in fellowship curricula, the flexibility from lack of standardization offers a wider array of possible experiences, which may benefit fellows with particular interests. We found that outdated publicly available information was very common; the time and effort spent in ascertaining what information was correct raises concerns that potential applicants may have difficulty as well.
